# Edible film based on corn zein containing dill extract and essential oil/β‐cyclodextrin inclusion complex: Shelf life enhancement of common carp fillet

**DOI:** 10.1002/fsn3.3353

**Published:** 2023-04-06

**Authors:** Ali Zolfaghari, Behnaz Bazargani‐Gilani, Narjes Aghajani

**Affiliations:** ^1^ Department of Food Hygiene and Quality Control Faculty of Veterinary Science Bu‐Ali Sina University Hamedan Iran; ^2^ Department of Food Science and Technology Bahar Faculty of Food Science and Technology Bu‐Ali Sina University Hamedan Iran

**Keywords:** common carp fillet, corn zein edible film, dill leaves' extract, encapsulated dill essential oil, shelf life

## Abstract

The aim of this study was to examine the impacts of corn zein edible film (Z) fortified with dill leaves extract (DE) and encapsulated dill essential oil with β‐cyclodextrin (nDEO) on the quality of refrigerated common carp fillet. Gas chromatography/mass spectrometry (GC/MS) analysis showed that the most frequent substances of DEO were apiol (35.1%) and carvone (31.4%), respectively. Designated treatments were as follows: (1) Control (C), (2) Z, (3) Z‐DE, (4) Z‐DEO, (5) Z‐nDEO, (6) Z‐DE‐DEO, and (7) Z‐DE‐nDEO. The physicochemical properties (thickness, moisture percent, tensile strength, elongation at break, Young's modulus, color, morphology, functional groups, and thermal resistance) of the activated films significantly improved (*p* ≤ .05). The total viable counts, lactic acid bacteria, *Enterobacteriaceae,* and psychrotrophic bacteria significantly decreased in all wrapped fillets compared to the unwrapped ones (*p* ≤ .05). Throughout storage period, the wrapped fillets exhibited lower changes in pH, thiobarbituric acid reactive substances, and total volatile‐based nitrogen values than the unwrapped fillets. According to the sensory findings, incorporating DE and nDEO in the zein films created significantly desirable aroma and flavor in the wrapped samples during storage time (*p* ≤ .05). Encapsulation of DEO with β‐cyclodextrin significantly fortified preservative effects of the films in fish fillets during storage period (*p* ≤ .05). In conclusion, the designated composite zein edible film containing DE and nDEO can be introduced as an active edible packaging in the shelf life improvement of common carp fillets during cold storage.

## Graphical abstract

The designated composite zein edible film containing dill extract and encapsulated dill essential oil can be introduced as an active edible packaging in the shelf life improvement of common carp fillets during cold storage.

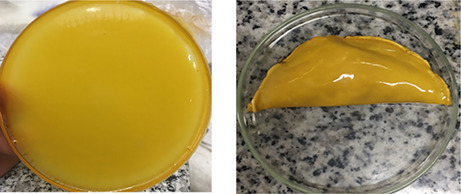

## 1 INTRODUCTION

Due to its high nutritional value, abundance, growth rate, and pleasant taste, the common carp (*Cyprinus carpio*) is known as one of the main edible freshwater fish species. High pH value, polyunsaturated fatty acids, and nonprotein nitrogen (NPN) compounds followed by fast microbial growth, oxidation, and autolytic reactions have decreased the shelf life of this desirable product (Moosavi‐Nasab et al., 2016). Active edible films and coatings with natural origin are the new approach for the shelf life enhancement of foods that have been recently preferred by consumers compared to synthetic approaches. This can be due to economic, health, and environmental issues. Zein protein is derived from corn and due to its unique features, such as filmmaking, significant thermal stability and gas barrier are used in food packaging. Zein edible films and coatings create a transparent yellowish, brilliant, and elastic appearance and show exceptional preservative features in food packaging (Barkhordari & Bazargani‐Gilani, 2021; Cui et al., 2020).

The inhibitory effects of edible films can be fortified by the incorporation of antimicrobial and antioxidant agents into them. Plant extracts and essential oils have been recently considered as natural preservatives. Dill with the scientific name of *Anethum graveolens* L. is an annual fragrant plant from Umbelliferae family and belongs to West Asia and Mediterranean. Previous studies demonstrated the presence of many unique antioxidant, antimicrobial, fragrant, and flavoring components in dill products. They found strong antimicrobial and antioxidant activities in dill essential oil and extract, respectively. Due to the unique sensory features (taste and aroma) of dill herb as well as its significant biochemical properties, simultaneous usage of DE and DEO can create a perfect and complete food additive (Singh et al., 2005; Tavakkoli et al., 2020).

However, the active compounds of volatile essential oils can be easily neutralized in the presence of environmental agents, such as oxygen, light, temperature, etc. (Dias Antunes et al., 2017; Rakmai et al., 2017). Furthermore, vigorous taste and aroma of essential oils may inappropriately affect the organoleptic features of foods. Also, fast evaporation and high water insolubility can limit the contact of essential oils with pathogens. Encapsulation by cyclodextrins can increase the stability and solubility of the essential oils in foods (Dias Antunes et al., 2017). Cyclodextrin molecules composed of the cyclic glucopyranosyl oligosaccharides linked by α‐(1,4) bonds have two unique structures consisting of a hydrophilic area and hydrophobic calyx, which can assemble inclusion complex with many substrates. Compatible cavity structure with common substrates, availability, and reasonable cost has caused β‐cyclodextrin to have the highest consumption among cyclodextrins (Dias Antunes et al., 2017; Rakmai et al., 2017).

Therefore, due to the long‐standing interest of Asians to consume dill plant along with common carp fillets, we intended to investigate the efficiency of zein edible film enriched with dill extract and essential oil/β‐cyclodextrin inclusion complex in the shelf life improvement of common carp fillet under refrigerated (4 ± 1°C) storage in this study.

## 2 MATERIALS AND METHODS

### 2.1 Extraction and analysis of DEO

*Anethum graveolens L*. seeds were provided by the local grocery of Hamedan. The seeds were powdered by a homemade mill (Hardston). The powdered seeds were extracted by hydro‐distillation method using Clevenger apparatus (Simax, Pyrexfan) for 4 h (Tavakkoli et al., 2020). The extracted DEO was collected in closed vials containing anhydrous sodium sulfate for dehydration and stored at refrigeration conditions until the next use. The ingredients of DEO were identified by a gas chromatograph (GC) equipped with a mass spectrometer (MS; Hewlett‐Packard).

### 2.2 Provision of β‐CD/DEO inclusion complex

Encapsulation of DEO by β‐CD was performed based on the method of Dias Antunes et al. (2017). After dissolving 2 g of β‐CD in distilled water (50 mL), 1.5 g of DEO was mixed with the solution under heating (35°C) and stirring (3 h). Then, the resulting solution was stored in the refrigerator for 24 h. The obtained precipitate in the solution was filtered through a Whatman No. 1 filter paper and then rinsed with ethanol (95%) and dried at 40°C for 24 h. The β‐CD/DEO inclusion complex was stored in the refrigerator (4 ± 1°C) until the next use.

### 2.3 Encapsulation efficiency

Encapsulation efficiency was determined based on the method of Bae and Lee (2008). Fifty milliliters of hexane was mixed with 2 g of IC and shaken for 2 min to extract free DEO. The obtained solution was then filtered through a Whatman No. 1 filter paper. After rinsing the remained powder with 20 mL of hexane solvent three times, it was dried at 60°C. The free DEO content was calculated by the weight difference of the inclusion complex powder before and after washing with hexane solvent. EE was computed by the following equation:

$$\text{Encapsulation efficiency}\;\left(\mathrm{EE}\right)=\frac{\text{Total}\;\mathrm{DEO}-\text{free}\;\mathrm{DEO}}{\text{Total}\;\mathrm{DEO}}\times 100$$

### 2.4 Extraction of DE

*Anethum graveolens L*. leaves were procured from the greengrocery. After washing and rinsing with potable water, the samples were dried in the shade at ambient temperature for 2 weeks. After grinding the dried leaves with the grinder (Hardston), the ratio of 1:10 of the sample to solvent [ethanol 70% (W/W)] was provided by the immersion method under shaking at 250 rpm for 24 h. After separating the solid and liquid phases by filtration through Whatman No. 1 filter paper, the liquid phase was concentrated by rotary evaporator apparatus (Lab Tech) at 40°C. Then, the remained solvent was evaporated by vacuum oven at 40°C. Then, the obtained extracts were stored at −18°C for the next tests (Singh et al., 2005).

### 2.5 Preparation of zein films

Corn zein powder (10% W/V) was dissolved in 96% ethanol and heated for 1 h at 80°C. Glycerol (20% W/V) and Tween 80 (0.2% W/V) were used as stabilizer and emulsifier agents in the films. Three percent of DE and DEO was added to the solutions of the desired films. The films were prepared by casting method so that the film solutions were poured on glass plates in an incubator chamber at 25°C with 50% relative humidity (RH). After drying, the formed films were gently peeled off. All prepared films were conditioned besides saturated magnesium nitrate for 48 h (RH of 50% at 25°C; Arcan & Yemenicioğlu, 2011).

### 2.6 Film analysis

#### 2.6.1 Tensile strength, elongation at break, and Young's modulus

Using a texture analyzer (Z 2.5, Zwick), the mechanical features, such as tensile strength (TS), elongation at break (EB), and Young's modulus (YM) of the films, were evaluated. The film specimens were considered with dimensions of 80 × 5 mm^2^. The crosshead speed was adjusted to 50 mm/min. At least, five points were measured for each specimen (ASTM, 2002).

#### 2.6.2 Thickness

Five different sites of the films were considered for thickness measurement using a digital micrometer (IP65 Alpa Exacto).

#### 2.6.3 Color

The color of the zein films was analyzed by a colorimeter (Minolta CR300 Series). The color indexes included L* (brightness/ darkness), a* (red/green), and b* (yellow/blue).

#### 2.6.4 Scanning electron microscopy

The morphological properties of the prepared films were determined by a scanning electron microscope (SEM; JEOL JSM‐840). The studied films were covered with gold and images were taken at 30 kV with a magnification of ×10,000.

#### 2.6.5 Moisture

The moisture content of the films was evaluated by drying small pieces of the films at 105°C for 24 h. The weight of the samples was considered before (W0) and after (W1) drying in the oven (Surendhiran et al., 2020). Moisture percent was computed as the weight difference of the samples before and after drying as follows:

$$\text{Moisture}\;\left(\%\right)=\frac{W0-W1}{W0}\times 100$$

#### 2.6.6 Fourier transform infrared spectroscopy

Fourier transform infrared spectrometer (FTIR; Helios) was used to identify functional groups and molecular interactions among zein, DE, DEO, and nDEO. A scan range from 4000 to 500 cm^−1^ at a resolution of 4 cm^−1^ was considered for the film analysis.

#### 2.6.7 Thermogravimetric analysis and differential scanning calorimetry

Thermal resistance of the studied films was determined by thermogravimetric analyzer (TGA) and differential scanning calorimetry (DSC; STA449F3, Netzsch). The temperatures were considered in the range of 25–500°C with a flow rate of argon at 20 mL/min and heating speed of 10°C/min (Surendhiran et al., 2020).

### 2.7 Preparation of the treatments

**FIGURE 1:**
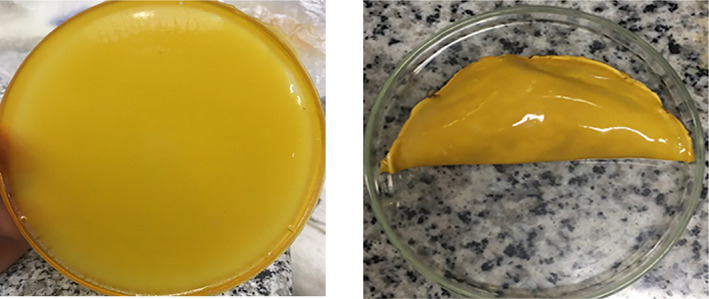
View of the prepared zein edible film (left side) and wrapped common carp fillet by zein film (right side).

Fresh common carp fillets were provided by a local fish processing unit immediately after hunting and shipped to the laboratory in the containers containing ice packs. Fish fillets were cut into 10 g pieces and wrapped in the prepared films by heat stitching (Figure 1). The treated fillets were packaged in polyethylene zip packs and stored in refrigerator (4 ± 1°C) for the next analyses. Microbial, chemical, and sensory analyses were carried out at 3‐day intervals to evaluate the shelf life and quality of the fish fillets for 12 days. The following treatments were studied: (1) Control (unwrapped fillets), (2) Z (wrapped fillets by zein films), (3) Z‐DE (wrapped fillets by zein films containing 3% DE), (4) Z‐DEO (wrapped fillets by zein films containing 3% DEO), (5) Z‐nDEO3% (wrapped fillets by zein films containing 3% nDEO), (6) Z‐DE3%‐DEO3% (wrapped fillets by zein films containing 3% DE and 3% DEO), and (7) Z‐DE3%‐nDEO3% (wrapped fillets by zein films containing 3% DE and 3% nDEO).

### 2.8 Microbial analysis

Total viable count, psychrotrophic bacteria, lactic acid bacteria (LAB), and *Enterobacteriaceae* populations of the samples were evaluated during storage days.

Fish fillet (10 g) was mixed with 90 mL of 0.1% sterile peptone water (Merck) in a sterile stomacher bag and stomached for 1 min. For microbial enumeration, 0.1 mL of serial dilutions of fillet homogenates was spread on the surface of agar plates. Total viable counts (TVC) were determined using Plate Count Agar (PCA, Merck) after incubation at 37°C for 24 h. Psychrotrophic bacteria were determined on Plate Count Agar and the plates were incubated at 7°C for 10 days. Lactic acid bacteria (LAB) were counted on de Man Rogosa Sharpe agar (MRS, QUELAB) incubated at 35°C for 2 days. *Enterobacteriaceae* were enumerated by the pour‐overlay method using Violet Red Bile Glucose (VRBG) agar (Merck). The plates were incubated at 37°C for 24 h. Three replicates of at least three appropriate dilutions depending on the sampling day were enumerated. The number of colony‐forming units was reported as Log_10_ CFU/g samples (Yousef et al., 2022).

### 2.9 Chemical analysis of fish fillet

#### 2.9.1 pH value

The pH value of the fillets was determined using a pH meter (Jenway). After homogenizing 5 g of the fish fillet with 25 mL of distilled water for 30 s, pH values of the obtained solutions were recorded (Brannan, 2008).

#### 2.9.2 TBARS value

Colorimetric technique was used for TBARS value determination based on the modified method of Pikul et al. (1989). TBARS value was recorded as milligram of malondialdehyde/kilogram of the sample. 1, 1, 3, 3‐tetraethoxypropane (TEP) was used for drawing a calibration curve.

#### 2.9.3 Total volatile basic nitrogen value

The distillation method using a Kjeldahl set (Pyrexfan) was used for total volatile basic nitrogen value (TVB‐N) value determination of the fish fillets based on the method of Fernández et al. (2009). TVB‐N values were calculated in milligram of nitrogen/100 g of the sample.

### 2.10 Sensory analysis

The sensory evaluation was performed using the 5‐point hedonic scale. Totally, 20 students of the Department of Food Hygiene and Quality Control participated in this test. They evaluated the odor, color, texture, and overall acceptability of the samples in each interval. Score 5 was considered an extremely liked sample and Score 1 was considered extremely disliked (Bazargani‐Gilani & Pajohi‐Alamoti, 2020; Tavakkoli et al., 2020).

### 2.11 Statistical analysis

Two replications of the research were carried out and all tests were run in triplicate. The collected data were reported as mean values ± standard deviations (SD). Variance analysis (ANOVA) with Tukey test was performed at the significance set of *p* ≤ .05 to compare differences among the treatments using SPSS software (IBM SPSS statistics 21).

## 3 RESULTS AND DISCUSSIONS

### 3.1 Gas chromatography–mass spectroscopy analysis of DEO components

Gas chromatography–mass spectroscopy (GC–MS) analysis of DEO identified 16 compounds, which was 98.746% of the total content. The main compounds of DEO were dill apiol (35.1%), carvone (31.4%), trans‐*p*‐Menth‐8‐en‐2‐one (10.160%), limonene (8.8%), and cyclohexanone (8.6%), respectively (Table 1).

**TABLE 1:** Chemical constituents of DEO.

Rank	Compound name	Composition %	RT^a^
1	Limonene	8.878	8.013
2	Cyclohexanone, 2‐methyl‐5‐(1‐methylethenyl), trans	8.600	11.213
3	*p*‐Menth‐8‐en‐2‐one, trans	10.160	11.352
4	2‐Cyclohexen‐1‐ol, 2‐methyl‐5‐(1‐methylethenyl), cis	0.440	11.724
5	trans‐Carveol	0.310	11.821
6	Cyclohexanol, 2‐methyl‐5‐(1‐methylethenyl)	0.398	11.979
7	Carvone	31.406	12.094
8	2‐Cyclohexen‐1‐one, 2‐methyl‐5‐(1‐methylethenyl)‐(R)	1.130	12.215
9	Anethole	0.039	12.887
10	Estragole	0.124	12.947
11	Thymol	1.018	13.084
12	Phenol, 2‐methyl‐5‐(1‐methylethyl)	0.412	13.194
13	Phenol, 5‐methyl‐2‐(1‐methylethyl)	0.071	13.230
14	1,3‐Benzodioxole, 4‐methoxy‐6‐(2‐propenyl)	0.373	16.653
15	Cycloheptasiloxane, tetradecamethyl	0.196	16.872
16	Apiol	35.191	18.189
Total identified (%)	98.746		

^a^ Retention time.

Tavakkoli et al. (2020) showed that the most frequent elements of DEO were alpha‐phellandrene (30.17%), limonene (28.31%), carvone (21.31%), dill ether (9.11%), p‐cymene (5.22%), and dill apiole (3.18%), respectively. In another study, the major ingredients of the dill herb essential oil were introduced as carvone (41.6%) and limonene (27.4%; Mutlu‐Ingok & Karbancioglu‐Guler, 2017). Rana and Blazquez (2014) found 12 compounds, constituting 96.7% of the DEO that included alpha‐phellandrene (31.8%), dill apiole (15.3%), dill ether (13.2%), limonene (11.8%), geraniol (10.6%), and *p*‐cymene (5.3%; Rana & Blazquez, 2014). Singh et al. (2005) found that the most frequent substance of DEO was carvone (55.2%) and limonene (16.6%), with dill apiole (14.4%) and linalool (3.7%) being the next ranks. These differences in the kind and percentages of the DEO constituents can be attributed to the essential oil extraction technique, herb drying method, plant age, climate, soil, harvesting season, and geographical position.

### 3.2 Physicochemical analysis of the films

#### 3.2.1 Thickness and moisture

**FIGURE 2:**
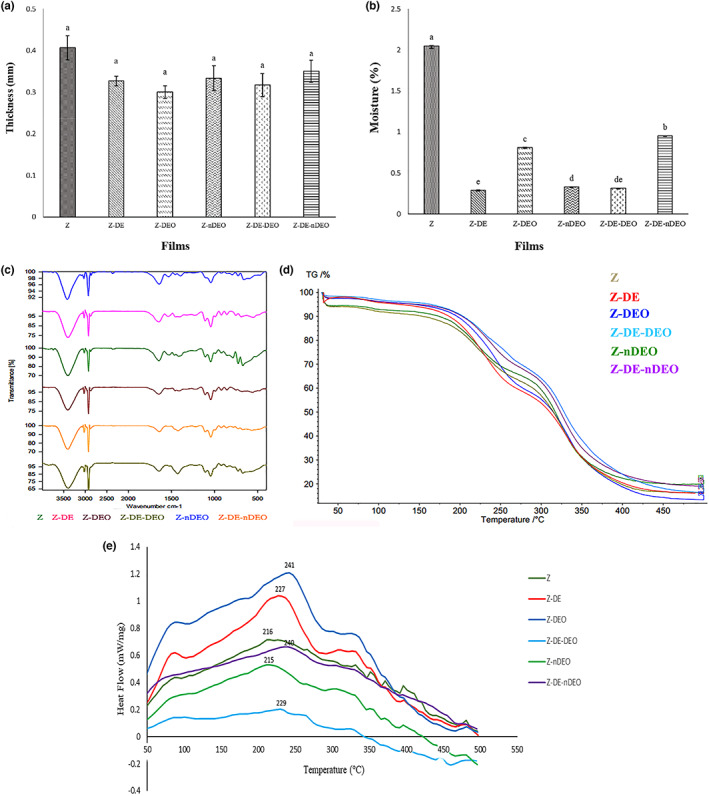
Physicochemical characteristics of the designated films: thickness (a), moisture (b), FT‐IR (c), TGA (d), and DSC (e).

The thickness of the studied zein films was in the range of 0.3–0.4 mm (Figure 2a). According to the obtained results, adding DE, DEO, and nDEO to the zein films decreased their thickness; but these changes were not statistically significant (*p* > .05). This can be due to the higher moisture content of the pure zein film compared to the others. According to Figure 2b, adding DE and DEO decreased moisture percent of the films which can be contributed to the decrease of the availability of their hydrophilic agents due to the interactions between zein polymer and DE, DEO, and nDEO. Also, these interactions can lead to the proper bonding between DE, DEO, nDEO, and zein polymer and as a result no their accumulations and thickness enhancement in the film (Nogueira & Martins, 2019; Wang et al., 2017). Furthermore, hydrophobic nature of DEO can cause increase and decrease in bonding to zein protein chains and water molecules, respectively, leading to the low moisture content of the zein films containing DEO (Arcan & Yemenicioğlu, 2011).

#### 3.2.2 Tensile strength, elongation at break, and Young's modulus

Mechanical characteristics of the edible films are very important, because they show the resistance of the film structure to elongation and rupture when submitted to tension. In order to package food, edible films must have proper flexibility and elasticity properties (Cui et al., 2020). Based on the results of Table 2, TS and EAB of Z‐DE and Z‐DEO films significantly (*p* ≤ .05) increased compared to the other films. Also, Z‐nDEO film showed the highest Young's modulus in comparison with the others and Z, Z‐DE‐nDEO, and Z‐DE‐DEO films were in the next ranks, respectively. Improvement of mechanical features of Z‐DE and Z‐DEO films can be correlated to the high concentration of their phenolic substances. These compounds have a lot of hydroxyl groups that can create hydrogen bonds with carbonyl groups of zein film that cause stronger interactions and surface adhesion among zein molecules; therefore, they modify the network of film matrix and improve its mechanical features. The oily nature of DEO could significantly (*p* ≤ .05) decrease the dryness and fragility of zein films and act as a softener (Liu et al., 2021; Salama et al., 2019). Furthermore, the hydrophilic groups of the phenolic compounds in DE can decrease the hydrophobic interaction (responsible for the low flexibility and brittleness in zein films) among zein molecules and therefore increase mobility and eliminate brittleness and flexibility issues of the films (Arcan & Yemenicioğlu, 2011; Liu et al., 2021). In previous studies, adding pomegranate peel extract to zein film significantly (*p* ≤ .05) increased TS and EAB values compared to the other films. They concluded that this improvement can be related to the viscous nature of pomegranate peel extract which can slightly increase the flexibility of zein film structure, proposing plasticizing and softening features (Cui et al., 2020; Mushtaq et al., 2018).

**TABLE 2:** Mechanical (stress, elongation at break, and young's modulus) and color (L*, a*, b*) properties of the studied films.

Films	Tensile strength (Mpa)	Elongation at break (%)	Young's modulus (Mpa)	L*	a*	b*
Z	0.91 ± 0.1^c^	30.68 ± 0.006^c^	74.8 ± 0.006^b^	71.62 ± 2.5^c^	7.34 ± 1.1^abc^	59.41 ± 3.7^a^
Z‐DE	1.56 ± 0.004^b^	54.57 ± 0.51^a^	39.04 ± 0.03^e^	78.03 ± 3.37^ab^	4.8 ±1.2^c^	55.49 ± 6.1^a^
Z‐DEO	1.59 ± 0.005^a^	40.27 ± 0.59^b^	40.04 ± 0.02^e^	77.01 ± 1.7a^b^	10.6 ± 0.9^ab^	58.79 ± 2.6^a^
Z‐nDEO	1.58 ± 0.01^b^	30.74 ± 0.008^c^	80.3 ± 1.1^a^	72.85 ±1.9^bc^	10.14 ± 1.9^a^	55.79 ± 4.1^a^
Z‐DE‐DEO	0.45 ± 0.006^d^	31.55 ± 0.003^c^	52.23 ± 0.6^d^	77.71 ± 1.8^ab^	6.2 ± 1.9^bc^	52.88 ± 9.8^a^
Z‐DE‐nDEO	0.48 ± 0.004^d^	30.78 ± 0.01^c^	62.86 ± 0.9^c^	80.44 ± 2.2^a^	5.68 ±1.7^c^	49.58 ± 11.2^a^

*Note:* Means within the same column (a, b, and c) with different letters are significantly different (*p* ≤  .05).

#### 3.2.3 Color

Film color is a determining factor in marketing, general appearance, and customer attraction (dos Santos Paglione et al., 2019). In this study, three color indexes of the zein films, including L* [brightness (+)/darkness (−)], a* [red (+)/green (−)], and b* [yellow (+)/blue (−)], were measured. According to Table 2, Z‐DE‐nDEO and Z films were significantly (*p* ≤ .05) the brightest and darkest films compared to the other treatments, respectively. Generally, incorporating DE, DEO, and nDEO into the zein film significantly (*p* ≤ .05) created brighter films than pure zein film. Adding green DE to the zein films significantly (*p* ≤ .05) decreased a* index in Z‐DE, Z‐DE‐nDEO, and Z‐DE‐DEO films among the others, respectively, that can be due to the green color of DE. Higher b* index of Z film can be due to the bold yellow color of zein polymer. Adding DE, DEO, and nDEO created light yellow appearance in the films but no significant differences were found in b* index of the studied films. Vahedikia et al. (2019) stated that adding chitosan nanoparticles and cinnamon essential oil to zein films significantly (*p* ≤ .05) decreased a* and b* values of the studied films, which could be due to the pale‐yellow color change of the zein films. Contrary to our findings, previous studies reported that decrease in L* value of the composite zein films containing essential oils can be related to the light scattering features of essential oils, creating an opaque appearance in the film color (Hosseini et al., 2015; Vahedikia et al., 2019). These differences can probably be related to the different original colors of the used essential oils and extracts in the films (Wang et al., 2017).

#### 3.2.4 Scanning electron microscopy

**FIGURE 3:**
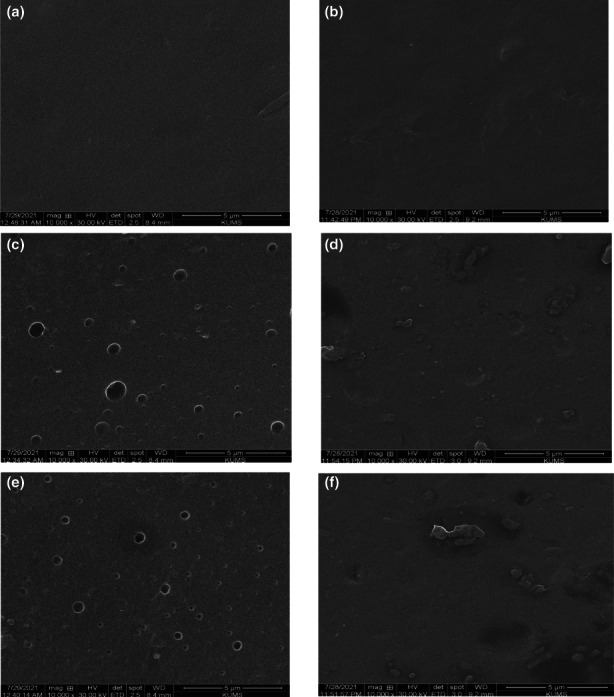
Scanning electron micrographs (SEM) of the designated films in magnifications of ×10,000. (a) Z, (b) Z‐DE, (c) Z‐DEO, (d) Z‐nDEO, (e) Z‐DE‐DEO, and (f) Z‐DE‐nDEO.

The scanning electron microscopy (SEM) graphs of the studied films are illustrated in Figure 3a–f. A smooth, homogeneous, and compact appearance was observed in the Z film (Figure 3a). The changes in the morphology of the films can be linked to the miscibility of the used materials. Z‐DE film showed more uniform and compact surface than Z film which can be correlated to the proper binding of DE components to the zein molecules (Figure 3b). Rubilar et al. (2013) reported that hydrophobic agents, such as carvacrol can create a sheet‐like and dense structure on the surface of the chitosan film so that their cross‐sectional images revealed the appearance of the stacked sheets in compact layers. The presence of the types of small and large cavities in Z‐DEO and Z‐DE‐DEO films can be attributed to the dispersed droplets of DEO in the zein matrix (Figure 3c,e; dos Santos Paglione et al., 2019). The observed irregular particles in Z‐nDEO and Z‐DE‐nDEO films revealed the presence of β‐CD/DEO inclusion complex (Figure 2d,f). Guimarães et al. (2015) observed the different size of rectangular‐shaped agglomerate crystals of β‐CD/carvacrol in their SEM images. Rakmai et al. (2017) found the irregular ridged‐shape particles in SEM images of hydroxypropyl‐beta‐cyclodextrin/guava leaf oil inclusion complex.

#### 3.2.5 Fourier transform infrared (FTIR) spectroscopy

Fourier transform infrared (FT‐IR) was used to identify the functional groups and the type of chemical reactions or bonding in the considered films. According to Figure 2c, zein films showed a broad peak at 3404 and two sharp peaks at 3027 and 2877 cm^−1^, attributed to the stretching vibration of N–H, O–H and H–C groups, respectively (Cui et al., 2020; Dong et al., 2017); two broad bands at 1646 and 1525 cm^−1^ were also linked to stretching vibration of C=O (amide I) and C–N functional groups (amide II), respectively (Cui et al., 2020; Dong et al., 2017; Ghamari et al., 2022). The bands situated at 1465 and 1377 cm^−1^ are ascribed to paired blending vibration with stretching one of CH_2_ and CH_3_, respectively, while the peaks at 1065 and 1045 cm^−1^ revealed the stretching vibration of ether C–O and primary alcohol C–O groups, respectively (Cui et al., 2020; Ghamari et al., 2022). Generally, peaks around 500–1500 cm^−1^ can be related to the existence of a functional group of polyphenols present in DE and DEO (Liu et al., 2021; Nunes et al., 2021; Zhou et al., 2021). As illustrated in Figure 2c, similar patterns of FT‐IR analysis of zein film were obtained in the other films. However, adding DE, DEO, and nDEO to the zein polymer created weaker peaks at higher frequencies compared to the pure zein film. This can be correlated to the interaction of these materials with zein molecules, and as a result, weakening the intermolecular bonds of the zein polymer. But simultaneous addition of DE and DEO to the zein film created stronger peaks at 3404 and 2877 cm^−1^ with lower frequencies compared to the free zein film.

#### 3.2.6 Thermogravimetric analysis

Thermal features of the studied films were analyzed by thermogravimetric analysis (TGA; Figure 2d) and DSC (Figure 2e). According to Figure 2d, the weight loss of all films happened at three steps: first, at 100–150°C due to the moisture, solvent, and volatile compounds’ loss in the zein films; second, at 150–270°C due to the decomposition of small functional groups with weak bounds; and third, at 270–400°C due to the complete destruction of the films. According to the graph, Z‐DE‐DEO film showed lower weight loss compared to the others at the same temperature and Z‐DE‐nDEO, Z‐DEO, Z‐nDEO, Z‐DE, and Z were in the next ranks, respectively. In other words, the simultaneous addition of DE, DEO, or nDEO to zein polymer caused the superior thermal resistance to free zein film. This phenomenon can be due to the potent interactions and intermolecular bonds among Z, DE, DEO, and nDEO and also higher boiling points of the molecules present in DE and DEO, resulting in higher thermal resistance of the studied films (Fazli et al., 2016; Surendhiran et al., 2020).

#### 3.2.7 Differential scanning calorimetry

Thermal behavior of the considered films was also shown using differential scanning calorimetry (DSC). In order to further study the variation of crystallinity, the DSC method was utilized to obtain the relevant thermal energy as a function of temperature. Generally, in DSC graph, an exothermic peak indicates an exothermic reaction caused by crystallization and the endothermic peak refers to an endothermic reaction by melting (Cui et al., 2020). The created exothermic peaks in the thermographs of the films cab were due to their transitions or reactions during the heating process. DSC thermograms of the studied films are illustrated in Figure 2e. According to the obtained results, by increasing the temperature, all the studied films showed an exothermic peak in certain temperatures that can be related to the crystallization phenomenon, and then graphs descended in the higher temperatures which can be due to the melting initiation. The exothermic peak of Z‐DEO treatment appeared at the highest temperature (241°C) among the others and Z‐DE‐nDEO (240°C), Z‐DE‐DEO (229°C), Z‐DE (227°C), Z (216°C), and Z‐nDEO (215°C) treatments were in the next ranks, respectively. It seems that adding the DEO and DE to zein film led to the formation of the proper molecular interaction and increasing crystallinity, following more thermal stability of them in comparison with the free zein films (Hu et al., 2015; Surendhiran et al., 2020). TGA and DSC findings demonstrated the excellent thermal stability up to 400°C of the combined films containing Z, DE, and DEO blends. In agreement with FT‐IR results, it seems that this formulation could fortify zein film structure.

### 3.3 Microbiological analysis

**FIGURE 4:**
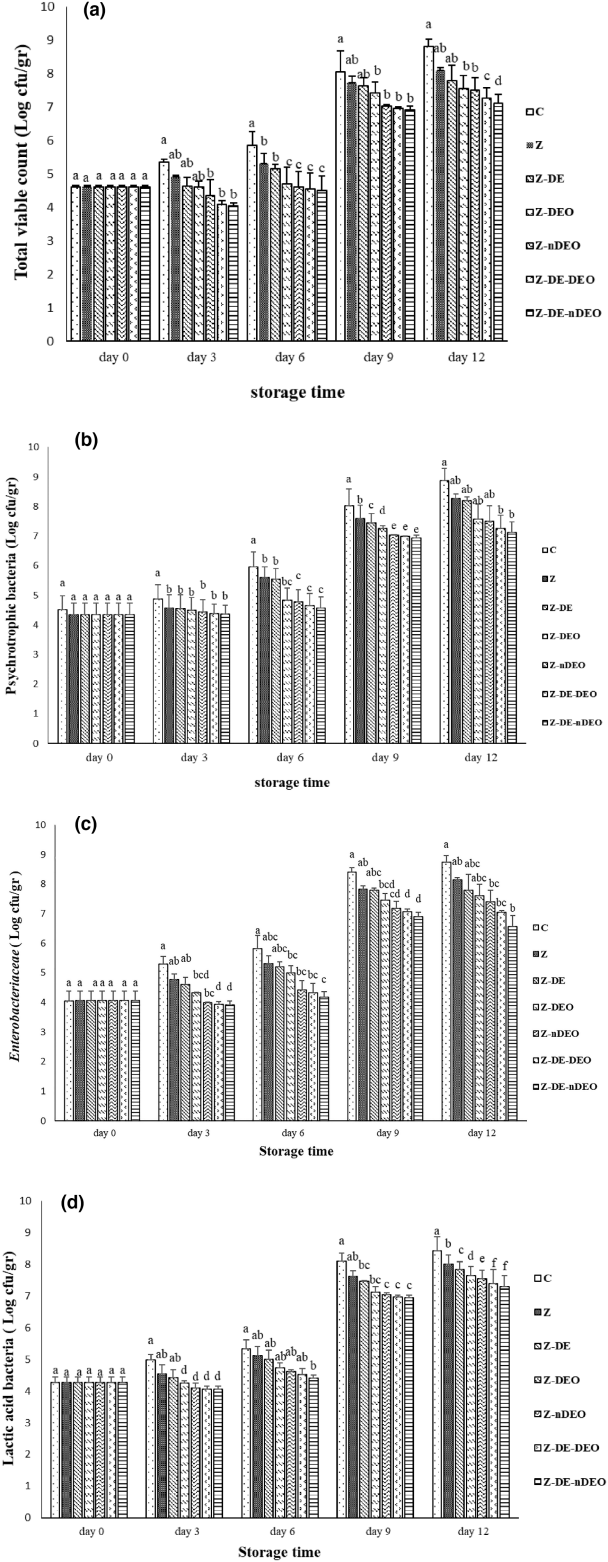
Average changes in microbial population [total viable count (a), psychotropic bacteria (b), *Enterobacteriaceae* (c), and lactic acid bacteria (d)] of the studied treatments during storage at 4°C. Different letters within the same interval (day) (a, b, c, etc.) indicate a statistically significant difference (*p* ≤ .05).

Figure 4a–d represents the changes in TVC, psychrotrophic bacteria, LAB, and *Enterobacteriaceae* of the treated common carp fillets during refrigerated storage. According to Figure 4a–d, no significant differences were found in initial bacterial populations, but an ascending trend was observed in all studied microbial groups of all treatments during storage time. The wrapped fillets with Z‐DE‐nDEO film showed the lowest microbial enumeration and Z‐DE‐DEO, Z‐nDEO, Z‐DEO, Z‐DE, Z, and control were in the next ranks during cold storage, respectively. β‐CD/DEO inclusion complex significantly (*p* ≤ .05) exhibited stronger antibacterial activities than free DEO which can be attributed to its high stability due to the protective effect of β‐CD, less evaporation, and controlled release compared to the free DEO (Dias Antunes et al., 2017). Previous research showed that carvone and limonene of DEO possessed significant antibacterial and antifungal activities. Also, DE, rich in linoleic acid, anethole, and dill apiole displayed a broad spectrum of antibacterial effects against the types of positive and negative gram bacteria (Singh et al., 2005).

### 3.4 Chemical analysis

**FIGURE 5:**
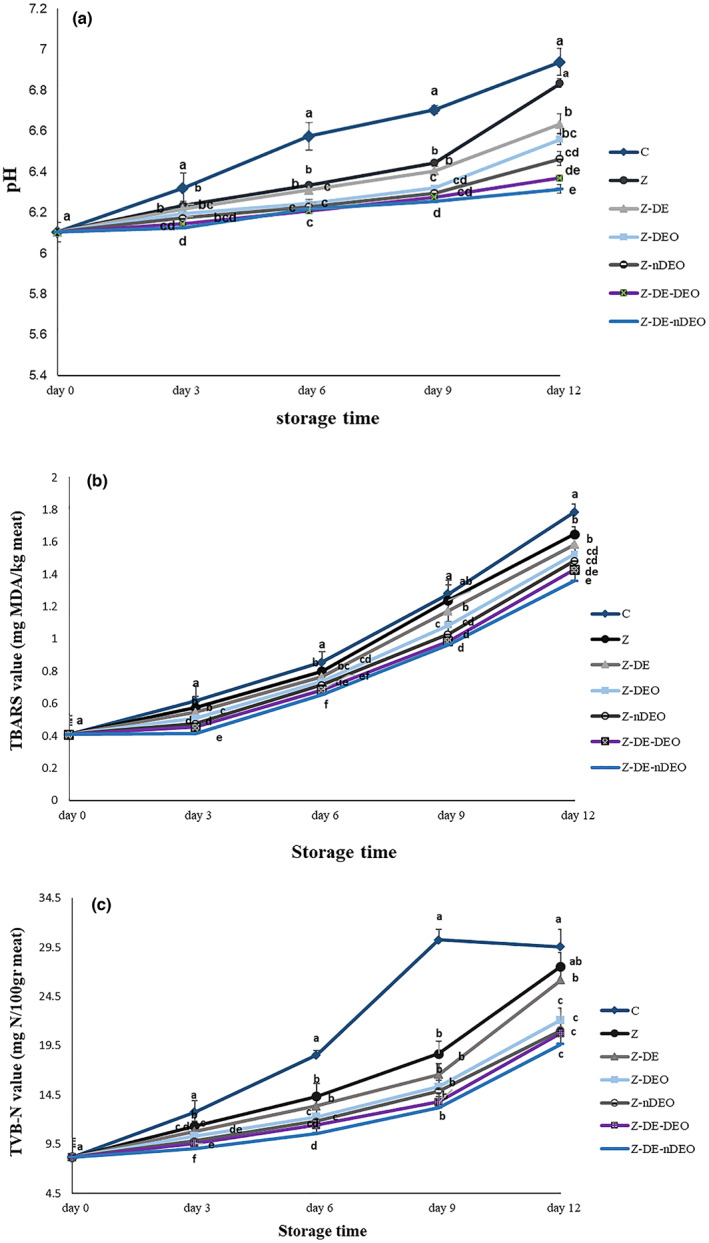
Average changes in chemical features [pH (a), TBARS (b), and TVB‐N (c) values] of the studied treatments during storage at 4°C. Different letters within the same interval (day) (a, b, c, etc.) indicate a statistically significant difference (*p* ≤ .05).

Figure 5a–c illustrates the changes of the chemical indexes [pH (A), TBARS (B), TVB‐N (C)] in the cold‐stored treated fillets. According to Figure 5a–c, no significant differences were found in initial pH, TBARS, and TVB‐N values of the fillets. All studied chemical indexes of the treatments continuously increased during storage time. In agreement with the pH values, by increasing the storage time, the production of volatile bases such as ammonia and trimethylamine significantly (*p* ≤ .05) increased; this can be related to the endogenous and exogenous (microbial) enzymes, such as protease and lipase that lead to the high TVB‐N value in the studied samples. Furthermore, lipase enzyme and autoxidation reaction of polyunsaturated fatty acids of the fish fillets produced peroxides and finally aldehyde compounds [e.g., malondialdehyde (MDA)] that caused TBARS index enhancement of the samples during prolonged storage (Barkhordari & Bazargani‐Gilani, 2021; Tavakkoli et al., 2020). In agreement with the previous research, wrapping fish fillets with edible films could significantly (*p* ≤ .05) control these chemical reactions that can be attributed to the decrease of microorganism growth, oxygen, and generally gas permeation into the used package (Surendhiran et al., 2020). Also, fortifying zein films by DE, DEO, and nDEO could significantly increase the protective effects of them that can be correlated to the presence of antimicrobial and antioxidant agents in these materials (*p* ≤ .05; Singh et al., 2005). Encapsulation of free DEO by β‐CD significantly improved these activities (*p* ≤ .05) so that Z‐DE‐nDEO film showed the best efficiency in decreasing chemical changes of the common carp fillets during storage period and Z‐DE‐DEO, Z‐nDEO, Z‐DEO, Z‐DE, and Z films were in the next ranks, respectively. Rakmai et al. (2017) observed that the encapsulation of yarrow essential oil in hydroxypropyl‐β‐CD (HPβCD) could inhibit the destructive effects of sunlight on its active compounds during 12 h. The encapsulation with HPβCD increased the yarrow essential oil stability by 27%–30%.

### 3.5 Sensory analysis

**FIGURE 6:**
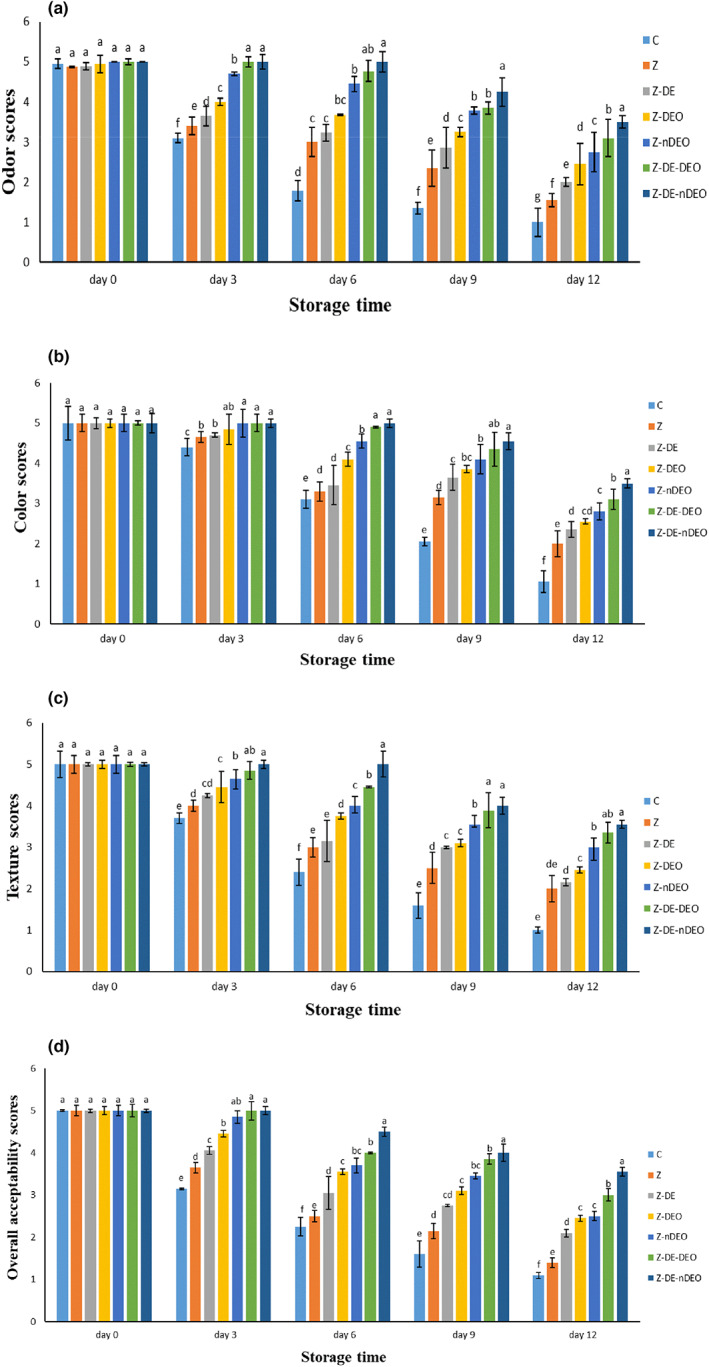
Average changes in sensory properties [odor (a), color (b), texture (c), and overall acceptability (d)] of the studied treatments during storage at 4°C. Different letters within the same interval (day) (a, b, c, etc.) indicate a statistically significant difference (*p* ≤ .05).

The sensory characteristics (odor, color, texture, and overall acceptability) of the fish fillets are illustrated in Figure 6a–d. No significant differences were observed in the primary sensory scores of the studied treatments and recorded in the range of 4–5. In accordance with the microbial and chemical analyses, unwrapped fillets (control group) earned unacceptable score (below 3) after 3 days of storage in terms of overall acceptability, odor, and texture features, while the used films significantly postponed loss of sensory scores in the wrapped fillets (*p* ≤ .05) so that Z‐DE‐nDEO and Z‐DE‐DEO treatments never got unacceptable scores during 12 days of the cold storage. This can be due to their protective effects against microbial and chemical changes of the samples, as well as creation of a pleasant sense in the panelists due to the unique taste and aroma of DE and DEO beside fish fillets (Barkhordari & Bazargani‐Gilani, 2021; Tavakkoli et al., 2020). Surendhiran et al. (2020) compared the effects of the chitosan film containing pomegranate peel extract (PE) with standard chemical preservative, such as sodium nitrite in the sensory assessment of the beef during storage period. They reported that PE exhibited similar performance to the sodium nitrite with acceptable scores. They concluded that this can be correlated to both antimicrobial and antioxidant activities of PE. In another study, sensory evaluation of zein‐coated breads showed a more pleasant texture than the uncoated samples during storage time. They reported that zein edible coating could significantly (*p* ≤ .05) retard staling in the studied breads during storage time compared to the control group (Mouzakitis et al., 2022).

## 4 CONCLUSION

It can be concluded that adding DE and DEO to zein edible film not only improved its mechanical features, thermal stability, and appearance but also successively upgraded the preservative effects (antioxidant and antimicrobial activities) of zein film in the shelf life enhancement of refrigerated common carp fillets. Also, the designated formulation created palatable sensory properties, including overall acceptability, odor, texture, and color in common carp fillets compared to pure zein films. Physicochemical activities of β‐CD/DEO inclusion complex significantly increased in comparison with free DEO by less evaporation, low movement into the food or bonding with food particles, and controlled release (*p* ≤ .05). Although the simultaneous addition of DE and nDEO to the designated Z film decreased its optimum mechanical properties, preservative effects of Z‐DE‐nDEO in shelf life enhancement of common carp fillet were very impressive. Therefore, considering the consumer demands for natural products, Z‐DE‐nDEO was introduced as the most promising biodegradable active film for food packaging, but in order to improve the mechanical properties of this film, using other techniques such as nanoemulsion for using DE and DEO in the films and other types of film production techniques, such as electrospinning method for the fabrication of this film is proposed for the future studies. Finally, releasing rates of DE, DEO, and nDEO from zein film need to be assessed.

## AUTHOR CONTRIBUTIONS

**Ali Zolfaghari:** Data curation (equal); formal analysis (equal); investigation (equal); methodology (equal); software (equal); writing – review and editing (equal). **Behnaz Bazargani‐Gilani:** Conceptualization (equal); data curation (equal); formal analysis (equal); investigation (equal); supervision (equal); validation (equal); writing – original draft (equal); writing – review and editing (equal). **Narjes Aghajani:** Conceptualization (equal); methodology (equal); resources (equal); software (equal); supervision (equal); writing – review and editing (equal).

## FUNDING INFORMATION

This research received no specific grant from any funding source.

## CONFLICT OF INTEREST STATEMENT

The authors declare that they have no conflict of interest.

## ETHICS STATEMENT

This study does not involve any human or animal testing.

## Data Availability

The data that support the findings of this study are available on request from the corresponding author.

## References

[fsn33353-bib-0001] Arcan, I. , & Yemenicioğlu, A. (2011). Incorporating phenolic compounds opens a new perspective to use zein films as flexible bioactive packaging materials. Food Research International, 44(2), 550–556. 10.1016/j.foodres.2010.11.034

[fsn33353-bib-0002] ASTM . (2002). Standard test method for tensile properties of thin plastic sheetingD882‐02. ASTM, Annual Book of American Standard Testing Methods.

[fsn33353-bib-0003] Bae, E. K. , & Lee, S. J. (2008). Microencapsulation of avocado oil by spray drying using whey protein and maltodextrin. Journal of Microencapsulation, 25(8), 549–560. 10.1080/02652040802075682 18465295

[fsn33353-bib-0004] Barkhordari, P. , & Bazargani‐Gilani, B. (2021). Effect of apple peel extract and zein coating enriched with ginger essential oil on the shelf life of chicken thigh meat. Journal of Food Measurement and Characterization, 15(3), 2727–2742. 10.1007/s11694-021-00863-4

[fsn33353-bib-0005] Bazargani‐Gilani, B. , & Pajohi‐Alamoti, M. (2020). The effects of incorporated resveratrol in edible coating based on sodium alginate on the refrigerated trout (*Oncorhynchus mykiss*) fillets’ sensorial and physicochemical features. Food Science and Biotechnology, 29(2), 207–216. 10.1007/s10068-019-00661-1 32064129 PMC6992829

[fsn33353-bib-0006] Brannan, R. G. (2008). Effect of grape seed extract on physicochemical properties of ground, salted, chicken thigh meat during refrigerated storage at different relative humidity levels. Journal of Food Science, 73(1), C36–C40. 10.1111/j.1750-3841.2007.00588.x 18211347

[fsn33353-bib-0007] Cui, H. , Surendhiran, D. , Li, C. , & Lin, L. (2020). Biodegradable zein active film containing chitosan nanoparticle encapsulated with pomegranate peel extract for food packaging. Food Packaging and Shelf Life, 24, 100511. 10.1016/j.fpsl.2020.100511

[fsn33353-bib-0008] Dias Antunes, M. , da Silva Dannenberg, G. , Fiorentini, Â. M. , Pinto, V. Z. , Lim, L.‐T. , da Rosa Zavareze, E. , & Dias, A. R. G. (2017). Antimicrobial electrospun ultrafine fibers from zein containing eucalyptus essential oil/cyclodextrin inclusion complex. International Journal of Biological Macromolecules, 104, 874–882. 10.1016/j.ijbiomac.2017.06.095 28652153

[fsn33353-bib-0009] Dong, S. , Gao, A. , Zhao, Y. , Li, Y.‐t. , & Chen, Y. (2017). Characterization of physicochemical and structural properties of atmospheric cold plasma (ACP) modified zein. Food and Bioproducts Processing, 106, 65–74. 10.1016/j.fbp.2017.05.011

[fsn33353-bib-0010] dos Santos Paglione, I. , Galindo, M. V. , de Medeiros, J. A. S. , Yamashita, F. , Alvim, I. D. , Ferreira Grosso, C. R. , Sakanaka, L. S. , & Shirai, M. A. (2019). Comparative study of the properties of soy protein concentrate films containing free and encapsulated oregano essential oil. Food Packaging and Shelf Life, 22, 100419. 10.1016/j.fpsl.2019.100419

[fsn33353-bib-0011] Fazli, Y. , Shariatinia, Z. , Kohsari, I. , Azadmehr, A. , & Pourmortazavi, S. M. (2016). A novel chitosan‐polyethylene oxide nanofibrous mat designed for controlled co‐release of hydrocortisone and imipenem/cilastatin drugs. International Journal of Pharmaceutics, 513(1–2), 636–647. 10.1016/j.ijpharm.2016.09.078 27693735

[fsn33353-bib-0012] Fernández, K. , Aspe, E. , & Roeckel, M. (2009). Shelf‐life extension on fillets of Atlantic Salmon (*Salmo salar*) using natural additives, superchilling and modified atmosphere packaging. Food Control, 20(11), 1036–1042. 10.1016/j.foodcont.2008.12.010

[fsn33353-bib-0013] Ghamari, M. A. , Amiri, S. , Rezazadeh‐Bari, M. , & Rezazad‐Bari, L. (2022). Physical, mechanical, and antimicrobial properties of active edible film based on milk proteins incorporated with Nigella sativa essential oil. Polymer Bulletin, 79(2), 1097–1117. 10.1007/s00289-021-03550-y

[fsn33353-bib-0014] Guimarães, A. G. , Oliveira, M. A. , Alves Rdos, S. , Menezes Pdos, P. , Serafini, M. R. , Araújo, A. A. , Bezerra, D. P. , & Quintans Júnior, L. J. (2015). Encapsulation of carvacrol, a monoterpene present in the essential oil of oregano, with β‐cyclodextrin, improves the pharmacological response on cancer pain experimental protocols. Chemico‐Biological Interactions, 227, 69–76. 10.1016/j.cbi.2014.12.020 25557507

[fsn33353-bib-0015] Hosseini, S. F. , Rezaei, M. , Zandi, M. , & Farahmandghavi, F. (2015). Bio‐based composite edible films containing *Origanum vulgare* L. essential oil. Industrial Crops and Products, 67, 403–413. 10.1016/j.indcrop.2015.01.062

[fsn33353-bib-0016] Hu, C. , Gong, R. H. , & Zhou, F. L. (2015). Electrospun sodium alginate/polyethylene oxide fibers and nanocoated yarns. International Journal of Polymer Science, 2015, 126041. 10.1155/2015/126041

[fsn33353-bib-0017] Liu, Z. , Lin, D. , Shen, R. , Zhang, R. , Liu, L. , & Yang, X. (2021). Konjac glucomannan‐based edible films loaded with thyme essential oil: Physical properties and antioxidant‐antibacterial activities. Food Packaging and Shelf Life, 29, 100700. 10.1016/j.fpsl.2021.100700

[fsn33353-bib-0018] Moosavi‐Nasab, M. , Shad, E. , Ziaee, E. , Yousefabad, S. H. , Golmakani, M. T. , & Azizinia, M. (2016). Biodegradable chitosan coating incorporated with black pepper essential oil for shelf life extension of common carp (Cyprinus carpio) during refrigerated storage. Journal of Food Protection, 79(6), 986–993. 10.4315/0362-028X.JFP-15-246 27296603

[fsn33353-bib-0019] Mouzakitis, C.‐K. , Sereti, V. , Matsakidou, A. , Kotsiou, K. , Biliaderis, C. G. , & Lazaridou, A. (2022). Physicochemical properties of zein‐based edible films and coatings for extending wheat bread shelf life. Food Hydrocolloids, 132, 107856. 10.1016/j.foodhyd.2022.107856

[fsn33353-bib-0020] Mushtaq, M. , Gani, A. , Gani, A. , Punoo, H. A. , & Masoodi, F. A. (2018). Use of pomegranate peel extract incorporated zein film with improved properties for prolonged shelf life of fresh Himalayan cheese (Kalari/kradi). Innovative Food Science & Emerging Technologies, 48, 25–32. 10.1016/j.ifset.2018.04.020

[fsn33353-bib-0021] Mutlu‐Ingok, A. , & Karbancioglu‐Guler, F. (2017). Cardamom, cumin, and dill weed essential oils: Chemical compositions, antimicrobial activities, and mechanisms of action against *Campylobacter* spp. Molecules, 22(7), 1191–1204. 10.3390/molecules22071191 28714890 PMC6152346

[fsn33353-bib-0022] Nogueira, D. , & Martins, V. G. (2019). Use of different proteins to produce biodegradable films and blends. Journal of Polymers and the Environment, 27(9), 2027–2039. 10.1007/s10924-019-01494-z

[fsn33353-bib-0023] Nunes, J. C. , Melo, P. T. S. , Lorevice, M. V. , Aouada, F. A. , & de Moura, M. R. (2021). Effect of green tea extract on gelatin‐based films incorporated with lemon essential oil. Journal of Food Science and Technology, 58(1), 1–8. 10.1007/s13197-020-04469-4 PMC7813924 33505046

[fsn33353-bib-0024] Pikul, J. , Leszczynski, D. E. , & Kummerow, F. A. (1989). Evaluation of three modified TBA methods for measuring lipid oxidation in chicken meat. Journal of Agricultural and Food Chemistry, 37(5), 1309–1313. 10.1021/jf00089a022

[fsn33353-bib-0025] Rakmai, J. , Cheirsilp, B. , Torrado‐Agrasar, A. , Simal‐Gándara, J. , & Mejuto, J. C. (2017). Encapsulation of yarrow essential oil in hydroxypropyl‐beta‐cyclodextrin: Physiochemical characterization and evaluation of bio‐efficacies. CyTA – Journal of Food, 15(3), 409–417. 10.1080/19476337.2017.1286523

[fsn33353-bib-0026] Rana, V. S. , & Blazquez, M. A. (2014). Chemical composition of the essential oil of *Anethum graveolens* aerial parts. Journal of Essential Oil Bearing Plants, 17(6), 1219–1223. 10.1080/0972060X.2014.894894

[fsn33353-bib-0027] Rubilar, J. F. , Cruz, R. M. S. , Silva, H. D. , Vicente, A. A. , Khmelinskii, I. , & Vieira, M. C. (2013). Physico‐mechanical properties of chitosan films with carvacrol and grape seed extract. Journal of Food Engineering, 115(4), 466–474. 10.1016/j.jfoodeng.2012.07.009

[fsn33353-bib-0028] Salama, H. E. , Abdel Aziz, M. S. , & Sabaa, M. W. (2019). Development of antibacterial carboxymethyl cellulose/chitosan biguanidine hydrochloride edible films activated with frankincense essential oil. International Journal of Biological Macromolecules, 139, 1162–1167. 10.1016/j.ijbiomac.2019.08.104 31415850

[fsn33353-bib-0029] Singh, G. , Maurya, S. , de Lampasona, M. P. , & Catalan, C. (2005). Chemical constituents, antimicrobial investigations, and antioxidative potentials of *Anethum graveolens* L. essential oil and acetone extract: Part 52. Journal of Food Science, 70(4), M208–M215. 10.1111/j.1365-2621.2005.tb07190.x

[fsn33353-bib-0030] Surendhiran, D. , Li, C. , Cui, H. , & Lin, L. (2020). Fabrication of high stability active nanofibers encapsulated with pomegranate peel extract using chitosan/PEO for meat preservation. Food Packaging and Shelf Life, 23, 100439. 10.1016/j.fpsl.2019.100439

[fsn33353-bib-0031] Tavakkoli, E. , Bazargani‐Gilani, B. , & Pajohi‐Alamoti, M. (2020). The impacts of tomato residuum extract with Arabic gum and dill essential oil on the shelf life improvement of trout fillets stored at chilly condition. Journal of Food Safety, 40(4), e12812. 10.1111/jfs.12812

[fsn33353-bib-0032] Vahedikia, N. , Garavand, F. , Tajeddin, B. , Cacciotti, I. , Jafari, S. M. , Omidi, T. , & Zahedi, Z. (2019). Biodegradable zein film composites reinforced with chitosan nanoparticles and cinnamon essential oil: Physical, mechanical, structural and antimicrobial attributes. Colloids and Surfaces B: Biointerfaces, 177, 25–32. 10.1016/j.colsurfb.2019.01.045 30703751

[fsn33353-bib-0033] Wang, L. , Wang, Q. , Tong, J. , & Zhou, J. (2017). Physicochemical properties of chitosan films incorporated with honeysuckle flower extract for active food packaging. Journal of Food Process Engineering, 40(1), e12305. 10.1111/jfpe.12305

[fsn33353-bib-0034] Yousef, A. E. , Waite‐Cusic, J. G. , & Perry, J. J. (2022). Analytical food microbiology: A laboratory manual (2nd ed.). Wiley.

[fsn33353-bib-0035] Zhou, Y. , Wu, X. , Chen, J. , & He, J. (2021). Effects of cinnamon essential oil on the physical, mechanical, structural and thermal properties of cassava starch‐based edible films. International Journal of Biological Macromolecules, 184, 574–583. 10.1016/j.ijbiomac.2021.06.067 34146564

